# Functional Analyses of Complement Convertases Using C3 and C5-Depleted Sera

**DOI:** 10.1371/journal.pone.0047245

**Published:** 2012-10-10

**Authors:** Marcin Okroj, Emelie Holmquist, Ben C. King, Anna M. Blom

**Affiliations:** Department of Laboratory Medicine, Lund University, The Wallenberg Laboratory, Malmö, Sweden; Radboud University Nijmegen Medical Centre, The Netherlands

## Abstract

C3 and C5 convertases are central stages of the complement cascade since they converge the different initiation pathways, augment complement activation by an amplification loop and lead to a common terminal pathway resulting in the formation of the membrane attack complex. Several complement inhibitors attenuate convertase formation and/or accelerate dissociation of convertase complexes. Functional assays used to study these processes are often performed using purified complement components, from which enzymatic complexes are reconstituted on the surface of erythrocytes or artificial matrices. This strategy enables identification of individual interactions between convertase components and putative regulators but carries an inherent risk of detecting non-physiological interactions that would not occur in a milieu of whole serum. Here we describe a novel, alternative method based on C3 or C5-depleted sera, which support activation of the complement cascade up to the desired stages of convertases. This approach allows fast and simple assessment of the influence of putative regulators on convertase formation and stability. As an example of practical utility of the assay, we performed studies on thioredoxin-1 in order to clarify the mechanism of its influence on complement convertases.

## Introduction

The complement system is a self-propagating, proteolytic cascade of proteins and functions within the framework of the innate immunity. In order to respond to multiple patterns of danger, complement can be initiated by three main pathways: classical, lectin and alternative. The first two are triggered upon detection of non-self or altered-self structures by sensor molecules able to recognize various molecular patterns (C1 complex, mannose-binding lectin (MBL) and ficolins) whereas the latter is constantly maintained active at a low level and propagated only due to lack of inhibition by the body’s own regulators [1], [2]. All pathways converge at the level of the C3 molecule, where downstream events can be amplified by a mechanism of positive feedback supported by complement convertases: the classical/lectin pathway C3 convertase (C4b2a) or the alternative pathway C3 convertase (C3bBb) [3]. These convertases further cleave C3 to C3b and C3a, of which C3b binds to nearby surfaces, providing a novel convertase assembly platform, or to pre-assembled C3 convertases, switching them to C5 convertases (C4b2aC3b or C3bBbC3b, respectively) [4]. The C5 convertase cleaves C5 molecules to C5a and C5b and the latter initiates formation of the membrane attack complex (MAC, C5b678polyC9) and its insertion into a target membrane. Osmotic lysis due to MAC deposition together with release of anaphylatoxins C3a and C5a as well as opsonization by C3b are the effector mechanisms of complement ensuring protection from invading pathogens, removal of immune complexes, dying cells and even orchestrating innate immune responses [1], [2]. However, complement may also harm own tissues when improperly controlled. The obvious need of keeping the system tightly balanced is reflected by the fact that, as well as 23 proteins recognized so far as engaged in the initiation and propagation of the complement cascade, almost the same number of complement inhibitors has been identified to date [1]. Any disturbance of this delicate balance [5] may result in increased susceptibility to infections [6], [7], [8], [9] or autoimmune diseases [10], [11], [12], [13], [14], [15] due to complement deficiency. Furthermore, excessive or misguided complement activation is involved in the majority of chronic and acute inflammatory diseases. Additionally, many bacteria and viruses have developed strategies to evade the complement system such as capturing host inhibitors or expressing their own efficient inhibitors, or secreting proteases which deplete complement (reviewed in [16]). The majority of described human as well as microbial complement inhibitors target complement at the stage of convertases. Most abundant fluid phase inhibitors present in serum at concentrations of several hundreds micrograms per millilitre such as factor H (FH) [17] or C4b-bidning protein (C4BP) [18] are characterized by convertase decay-acceleration activity, an ability to accelerate convertase disassembly, as well as cofactor activity, as they act as cofactors supporting cleavage by factor I (FI) of the activated complement components C3b and/or C4b necessary for convertase formation. Furthermore, all human cells express at least one membrane-bound inhibitor displaying decay-acceleration activity (CD35/CR1, CD55/DAF) or cofactor activity (CD35/CR1, CD46/MCP) [1]. Functional studies of acknowledged and putative complement inhibitors and dissecting their influence on convertase formation and disassembly are crucial for assessment of their overall importance in the whole complement cascade.

Historically, assays determining decay accelerating activity were performed on antibody sensitized sheep erythrocytes (classical pathway) or rabbit erythrocytes (alternative pathway) in veronal buffers permissive for the individual pathways [19], [20], [21]. DGVB^++^ buffer containing magnesium and calcium ions necessary for C2–C4 interaction [22] and C1q complex formation [23], respectively, enables activation of the classical and the lectin pathways as well as the alternative pathway (in sufficiently high serum concentrations). Only the alternative pathway can proceed in Mg^2+^-EGTA buffer, containing magnesium ions and a calcium chelating agent [24]. In a typical assay, the classical C3 convertase is built stepwise on erythrocytes using purified C1, C4 and C2 and termed EAC142 (E stands for sheep erythrocytes and A for amboceptor; sensitizing antibody). Further incubation with purified C3 results in classical C5 convertase formation (EAC1423). The same platform can be used to build functional alternative convertases. In such case C4 and C2 are dissociated from the complex by incubation in a buffer containing 10 mM EDTA followed by addition of factor D, factor B (FB) and properdin [19]. Studies of the alternative convertase were also performed using microplate methods, where purified C3b (or C3b dimers for C5 alternative convertase) were coated on the surface and followed by incubation with FB and D in the presence of nickel ions. The remaining convertase was then quantified by detection of bound FB [19]. Another method successfully used to determine the mode of complement inhibition of the alternative complement convertase employed surface plasmon resonance techniques [25].

**Figure 1:**
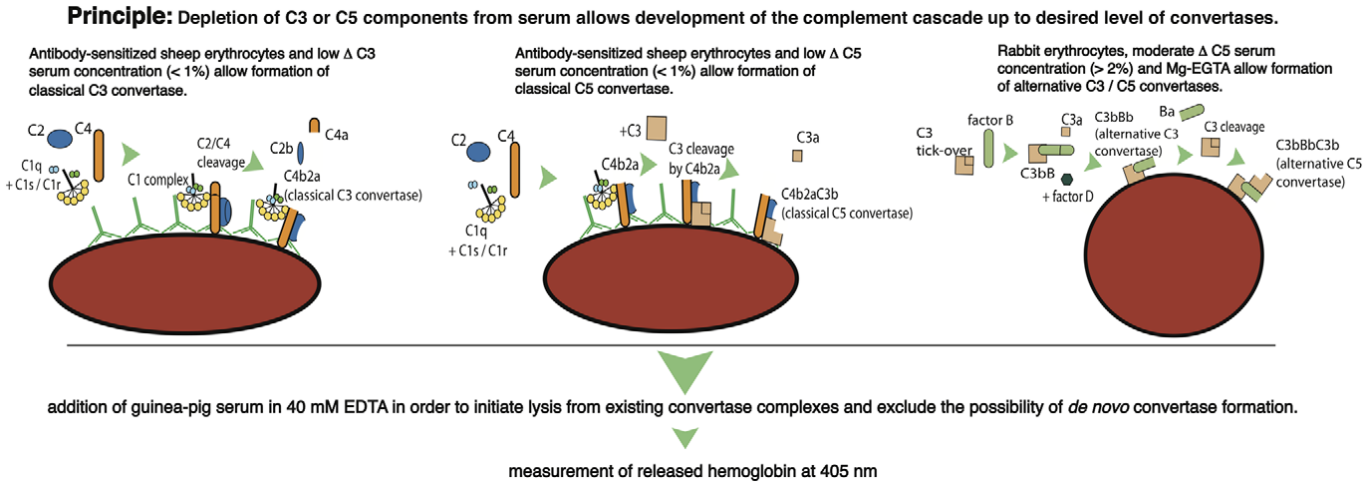
Principles of novel complement convertase assay. Classical complement convertases are assembled on sheep erythrocytes sensitized with antibodies and incubated with C3 or C5-depleted serum. The lack of a particular component allows the complement cascade to proceed only to the stage of classical C3 convertase (C4b2a) or classical C5 convertase (C4b2a3Cb). Low serum concentration and sensitization with antibodies enable the classical complement pathway. Formation of alternative C3 (C3bBb) and C5 (C3bBbC3b) convertases is performed on rabbit erythrocytes upon treatment with moderate concentration of C5-depleted serum in Mg –EGTA buffer (sequestering calcium but not magnesium). Addition of guinea pig serum (as a source of missing complement component as well as MAC components) diluted in EGTA initiates lysis only from preformed convertase complexes since chelating of divalent cations prevents disables de novo convertase formation. Therefore, the amount of released hemoglobin is proportional to the initial number of active convertases formed on erythrocytes.

Here we propose a novel method based on hemolytic assays and C3 or C5-depleted sera. Such sera, having C3 or C5 removed by affinity chromatography, support development of the complement cascade up to the desired convertase level. It eliminates the need of costly, stepwise and time consuming formation of enzymatic complex from purified components. Also, usage of whole serum limits the risk of protein inactivation during the procedure and gives much more physiological conditions than those requiring protein immobilization on an artificial surface or heavy metal ions. Building complement convertases on erythrocytes enables an easy readout of the assay by spectrophotometric quantification of released hemoglobin. The principles of the method are shown and explained in Fig. 1.

## Methods

### Ethics Statement

Human serum and plasma were prepared from blood of healthy volunteers after written informed consent had been obtained with the specific permit by the ethics committee of Lund University (permit number 418/2008).

### Sera, Proteins and Antibodies

C3 and C5-depleted sera as well as function blocking antibody against complement FI (#1; A247) were purchased from Quidel. Purified complement components C1 inhibitor, C3, C5, C6, C7, C8, C9 and FB were purchased from Complement Technologies. Guinea pig serum was from Harlan Laboratories. Antibody MK54 against protein S (control antibody in function blocking experiments) and MK104 antibody against C4BP, which binds to its N-terminal part and blocks cofactor activity were home made and purified using protein A affinity chromatography as described [26], [27]. Antibody OX24, which inhibits the interaction of FH with surface bound C3b [28] was obtained from hybridoma cells purchased from Health Protection Agency Culture Collections and purified from conditioned medium by binding to 5 ml protein G-sepharose column (GE Healthcare) following washing with PBS and elution with 0.1 M glycine pH 2.5. CD55 (DAF) and Coxsackie adenovirus receptor (CAR) were cloned into the pTorsten vector (kind gift of Dr. Brad Spiller, Cardiff University) in frame with C-terminal human Fc tag and expressed in CHO cells, as described [29]. C4BP [30], FI [31] and FH [32] were purified from human plasma as described previously and thioredoxin-1 (Trx-1) as well as its active site mutant CC/SS variant (Δ Trx-1) were produced and purified as described [33]. Trx-1 and Δ Trx-1 as well as BSA (Fraction V, pH 5.2, Sigma) used in convertase formation assays were first preincubated with DTT at a final concentration of 25 mM for 15 min at 37°C.

### Classical Convertase Assays

Sheep erythrocytes (E) were purchased from Hatunalab AB. E (3.75×10^8^ corresponding to 150 µl of stock solution) were washed with DGVB^++^ buffer (2.5 mM veronal buffer, pH 7.3, 72 mM NaCl, 140 mM glucose, 0.1% gelatin, 1 mM MgCl_2_, and 0.15 mM CaCl_2_), pelleted and mixed with 1 ml of DGVB^++^ containing amboceptor (A; Complement Technologies) diluted 1∶1000. Samples were then incubated for 10 min at 37°C with 210 rpm shaking. Afterwards EA were washed twice, pelleted and resuspended in 500 µl of DGVB^++^. To ensure the use of an equal number of EA in each experiment, EA suspensions were adjusted so that 10 µl of such suspension lysed with 90 µl of water had an absorbance at 405 nm of 2.0.

For assessment of *Tmax* (the time point at which the maximal number of active convertase complexes are present on the cell surface), 10 µl of EA were placed in wells of a 96-well V-shape microplate (Nunc) and 40 µl of DGVB^++^ buffer containing the given concentration of C3 or C5-depleted sera was added at different time points in order to assemble convertases. The plate was kept in a Thermomixer Comfort (Eppendorf) at 30°C with 600 rpm shaking during the whole incubation period. To initiate complement mediated lysis from existing convertase complexes, 50 µl of 40 mM EDTA-GVB buffer (40 mM EDTA, 5 mM veronal buffer, 0.1% gelatin, 145 mM NaCl) containing 1∶40 guinea pig serum was added and the plate was incubated for another 20 minutes at 37°C and 600 rpm. Alternatively, lysis was developed with a mix of purified C5–C9 components (10 µg/ml each) with or without C3 (20 µg/ml) in 40 mM EDTA-GVB buffer. Cells were pelleted and 80 µl of the supernatant containing hemoglobin released from lysed EA were measured at 405 nm in a microplate reader (Cary50Bio, Varian). Blocking of soluble complement inhibitors in the Tmax assay was achieved by addition of function blocking antibodies to serum prior to mixing with EA: OX24 at a final concentration of 200 nM, MK104 at 240 nM and #1/A247 at 14 nM. These antibodies were tested both separately and in combination and compared to the control antibody MK54 at the same concentration. For testing each blocking antibody separately, we chose an incubation time of 30 minutes.

The effect of complement inhibitors on formation and decay-acceleration of the classical C3 and C5 convertases was studied at 0.5% C3 or C5-depleted serum, respectively. Addition of a putative complement inhibitor together with depleted serum allows study of concomitant action of an inhibitor on both formation and dissociation of convertases. On the other hand, incubation of an inhibitor with cells already bearing formed convertase allows the assessment of the inhibitor’s ability to accelerate convertase decay. The classical C3 convertase was assembled for 5 minutes with or without complement inhibitors depending on the aim of experiment. Afterwards EA were washed with 200 µl of DGVB^++^ buffer and centrifuged 1 min at 800×G. In the convertase formation assay, this step was followed by development of complement mediated lysis from existing convertase complexes. EA were diluted in 50 µl DGVB^++^ and an additional 50 µl of 40 mM EDTA-GVB buffer containing guinea pig serum and incubated as described for *Tmax* assessment. In the decay-acceleration assay, cells with pre-assembled convertases were diluted in 50 µl DGVB^++^ containing the protein to be studied and incubated for additional 10 minutes at 30°C. Afterwards, EA were washed and complement mediated lysis was assessed. Assays on the classical C5 convertase were performed in the same way but the convertase was assembled for 2.5 minutes.

### Blocking of FB Activity in Classical C5 Convertase Assay

In order to assess contribution of the alternative convertases as an amplification loop of the classical pathway, we blocked FB activity during and after formation of the classical C5 convertase. EA were mixed with 40 µl DGVB^++^ containing C5-depleted serum at a final concentration of 0.5% and 60 µg/ml of function blocking mouse anti human FB antibody (Quidel, A227) or control antibody MK54 against human protein S, and then the C5 convertase was allowed to assemble according to the standard scheme used in previous assays. Then EA were washed and the same amount of anti-FB antibody diluted in 50 µl of DGVB^++^ was added and incubated as in the decay-acceleration assay before development of complement-mediated lysis by addition of guinea pig serum in 40 mM EDTA-GVB buffer.

### Alternative Convertase Assays

Rabbit erythrocytes (rE) suspended 1∶1 in Alsever’s solution (114 mM glucose, 28 mM Na-citrate dihydrate, 68 mM NaCl, 0.2 mM citric acid) were washed with Mg^++^EGTA buffer (2.075 mM veronal buffer pH 7.3, 10 mM EGTA, 7 mM MgCl_2_, 0.083% gelatin, 116 mM glucose, 60 mM NaCl) until there was no visible hemoglobin in the supernatant. Then, 3.75×10^8^ of rE (corresponding to 150 µl of Alsever’s solution) were suspended in 1 ml of Mg^++^EGTA and used as a master stock for the assays. To ensure an equal number of rE in each experiment, rE suspensions were standardized so that 10 µl of such suspension lysed with 90 µl of water had an absorbance of 1.0 at 405 nm.

For assessing *Tmax*, 10 µl of rE were placed in wells of a 96-well V-shape microplate (Nunc) and 40 µl of Mg^++^EGTA buffer containing a given concentration of C5-depleted serum was added at different time points in order to assemble convertases. Complement-mediated lysis resulting from assembled convertase complexes was initiated and measured as described for the classical pathway assays. Blocking of soluble complement components was performed in the same way as during the assessment of Tmax for classical convertases but the final concentration of function-blocking antibodies was increased three times and the incubation time for testing each blocking antibody separately was 60 minutes.

Determinations of the influence of a given protein on alternative convertase formation and decay acceleration were performed according to the same principle as for the classical pathway. However, 5% C5-depleted serum in Mg^++^EGTA was used, formation of convertase was allowed for 20 minutes and the dissociation time was set to 5 minutes. Blocking of FH activity (applied for Trx-1 experiments) was done with 400 nM of OX24 antibody.

### Determination of Soluble Complement Inhibitors in serum

The levels of FH, FI and C4BP in C3 and C5-depleted sera were measured by ELISA as described [34].

Determination of C3a production was performed by western blotting. Rabbit erythrocytes were incubated with 50 µl of 5% C5-depleted serum and then samples were centrifuged and supernatant collected. After addition of 3× Laemli sample buffer with DTT, 5 µl of the sample was boiled for 5 minutes and loaded onto 13.5% SDS-PAGE, then transferred to a PVDF membrane and probed with mouse anti-human C3a/C3a des-Arg monoclonal antibody (Hycult) diluted 1∶500 followed by anti-mouse HRP-conjugated antibody (Dako Cytomation) diluted 1∶1000. The intensity of the C3a band in each sample was evaluated with MultiGauge 2.0 software (Fuji Film).

## Results

### Determination of Functional Depletion of C3 and C5 from serum

**Figure 2:**
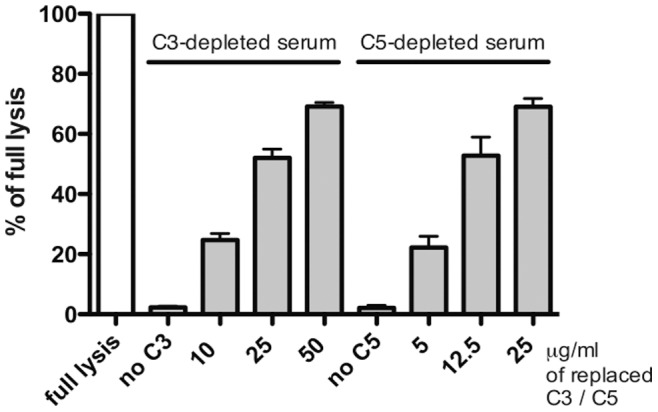
Testing of functional depletion of C3 or C5 from serum. EA were diluted in DGVB^++^ buffer and incubated with 0.5% of C3 or C5 depleted serum supplemented or not with purified C3 or C5, respectively. Results are collected from three independent experiments.

Effective depletion of C3 or C5 from serum is a crucial parameter in our assay and such sera should not cause any hemolysis unless supplemented with the missing complement component. Therefore we subjected EA to 0.5% of each serum and measured their hemolytic activity. Hemolytic activity was lacking from the C3 and C5 depleted sera but was restored after supplementation with purified C3 or C5, respectively, in a dose dependent manner (Fig. 2).

### Evaluation of Tmax of the Classical and the Alternative Convertases

**Figure 3:**
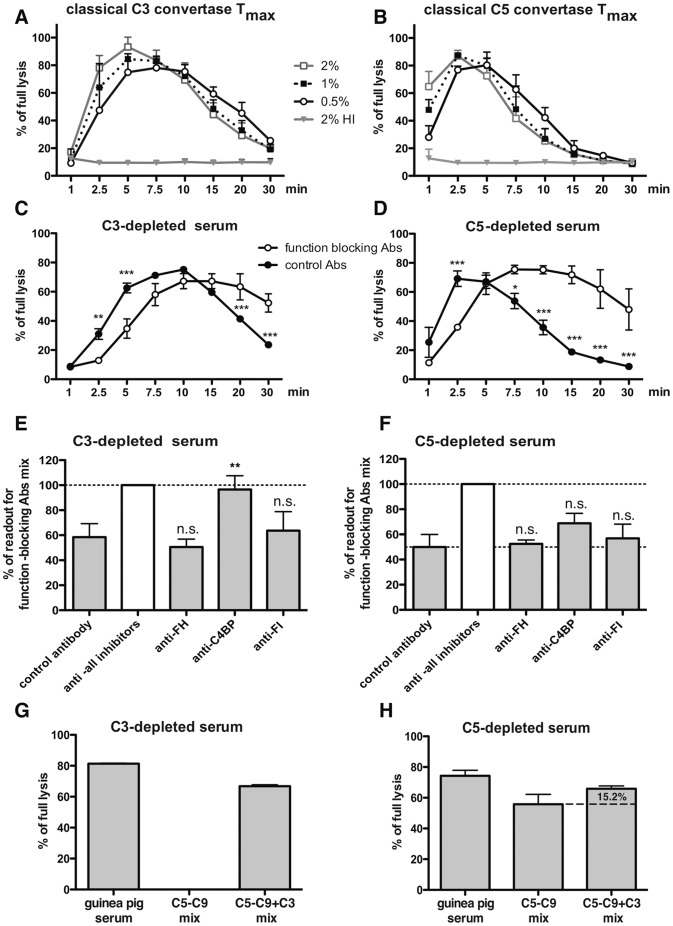
Tmax of classical convertases. EA were incubated with C3-depleted serum (A, C,) or C5-depleted serum (B, D) for indicated time periods and thereafter complement mediated lysis was developed with guinea-pig serum in a buffer containing EDTA, thus preventing *de novo* assembly of convertases. Values were collected from three independent experiments and related to full lysis of EA. In panels C and D, EA were incubated with a mix of function blocking antibodies against soluble complement inhibitors FH, C4BP and FI or control antibody against protein S. Symbols * and *** stand for p<0.05 or p<0.001, according to two-way ANOVA. Panels E and F show the effect of the individual function-blocking antibodies measured at 30 minutes. The symbol ** stands for p<0.01 according to one-way ANOVA. Panels G and H show readouts of classical C3 (G) and C5 (H) convertase assays (performed in triplicates) when developed at their *Tmax* with guinea-pig serum or a mix of purified C5–C9 complement components with or without C3.

Complement convertases exhibit both spontaneous and catalytic decay and these processes are dependent on several external factors such as pH, temperature and presence of other complement factors [35], [36]. In order to understand the dynamics of convertase formation and to evaluate the optimal time and serum concentration for further assays, we investigated the activity of convertases over time. Convertase formation was allowed for increasing time periods and then guinea pig serum diluted in EDTA, in order to prevent further convertase assembly, was added as a source of MAC components. The classical C3 convertase was evaluated using C3 -depleted serum concentrations ranging from 0.5 to 2% and showed maximal efficiency when the time of formation was set between 5 to 10 minutes. *Tmax* was reached faster at higher serum concentrations but at higher serum concentrations we also observed faster convertase decay (Fig. 3a). These results suggest that soluble inhibitors present in serum are at least partially responsible for convertase decay and indeed functional convertase complexes persisted longer when convertase formation was performed in the presence of a mix of function-blocking antibodies directed against inhibitors as compared to formation with control antibodies (Fig. 3c). Out of three soluble complement inhibitors, C4BP turned out to be most relevant for the C3 clasical convertase (Fig. 3e). The same effect was observed in the classical C5 convertase assay (performed with C5-depleted serum at the same concentration range as for C3-depleted serum in the C3 convertase Tmax assay) but *Tmax* was reached between 2 and 5 minutes followed by a fast decay (Fig. 3b,d). Notably, there was no single complement inhibitor most relevant for the decay of the classical C5 convertase but rather they have a synergistic effect (Fig. 3f). Differences in *Tmax*/decay timing between C3 and C5-depleted sera could be due to differences in the content of complement factors and inhibitors. However, C4BP, FI and FH concentrations measured by ELISA were always higher in the C3-depleted serum (Table 1) despite the fact that decay was faster in the C5-depleted serum. This, combined with the higher effectiveness of inhibitor-blocking antibodies in the C5-depleted serum (Fig. 3d) suggests that the classical C5 convertase is more prone to disassembly by soluble complement inhibitors as compared to the classical C3 convertase.

**Table 1:** Concentration of soluble complement inhibitors in depleted sera (µg/ml ± standard deviation).

Complement inhibitor	Δ C3 serum	Δ C5 serum
C4BP	283.3±46.0	227±29.3
FI	34.2±1.4	24.8±1.7
FH	257.0±11.2	202.2±14.8

Reference values in serum, according to [40]: FI: 35 µg/ml, C4BP: 250 µg/ml, FH: 500 µg/ml.

In order to examine whether obtained readouts are solely due to C3 or C5 convertase activity, we used a mix of purified C5–C9 proteins instead of guinea pig serum to develop the assay. The C5–C9 mix was ineffective in the development of the classical C3 convertase assay unless supplemented with C3, therefore once more confirming the functional elimination of C3 from C3-depleted serum (see Fig. 1) and thus verifying a readout specific for the C3 convertase (Fig. 3g). Conversely, application of a C5–C9 mix to develop the classical C5 convertase assay should result in hemolysis, and it did so (Fig. 3h). However, addition of purified C3 to the C5–C9 mix indicated that approximately 15% of the readout was due to C3 convertase activity (Fig. 3h) and a similar percentage of unspecific effect may be expected when guinea pig serum is used.

**Figure 4:**
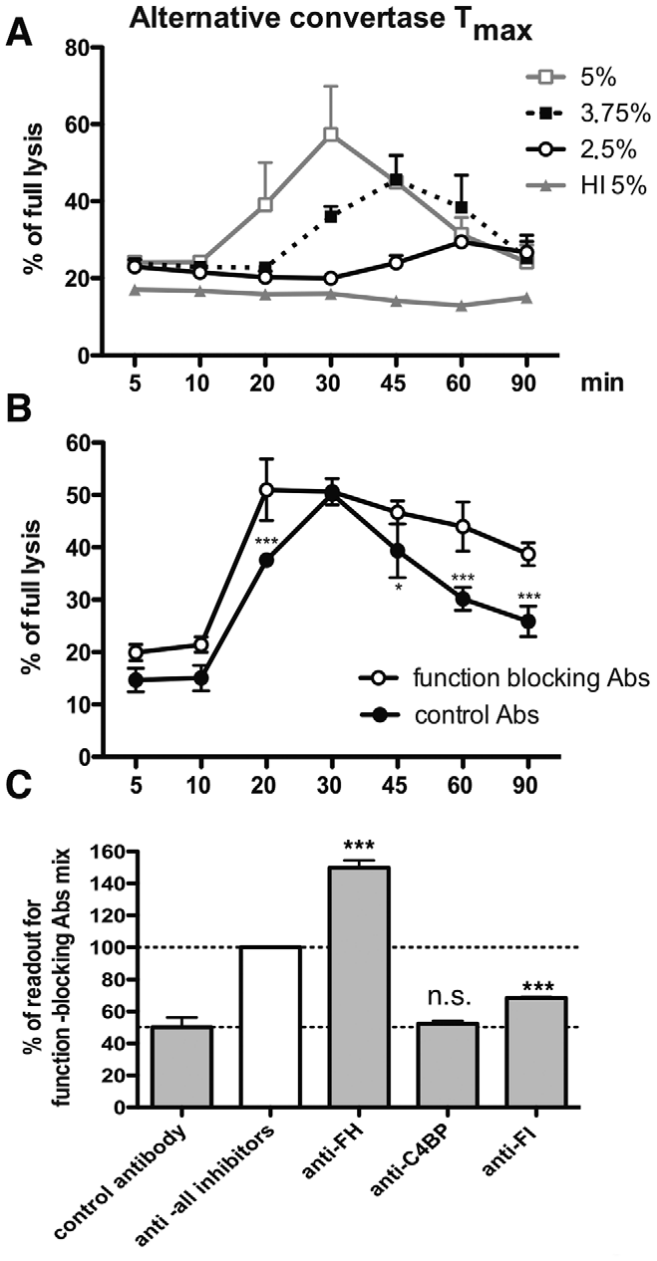
Tmax of alternative convertases. rE were incubated with the C5-depleted serum for indicated time periods and thereafter complement mediated lysis was developed with guinea-pig serum in buffer containing EDTA (A). Values were collected from three independent experiments and related to full lysis of EA. In panel B, rE were incubated with a mix of function blocking antibodies against soluble complement inhibitors FH, C4BP and FI or control antibody against protein S. Symbols * and *** stands for p<0.05 or p<0.001, according to two-way ANOVA. Panel C shows the effect of individual function-blocking antibody measured at 60 minutes. Symbol *** stands for p<0.001 according to one-way ANOVA.

Alternative convertase formation was tested within the concentration range of 2.5 to 5% serum. We observed the same trend as was seen for its classical counterpart, i.e. faster *Tmax* and faster decay at higher serum concentrations. However, the maximal activity was achieved as late as after 30 minutes at 5% serum concentration and even 60 minutes at 2.5% serum (Fig. 4a). Blocking of soluble complement inhibitors resulted, similarly to classical convertase assays, in longer persistence of functional convertases, but opposed to classical convertases, in a more rapid *Tmax* (Fig. 4b) instead of delayed *Tmax* (Fig. 3c,d). As a possible explanation of these differences, we can offer consumption of complement components due to classical pathway initiation outside of the EA. It might take place on immunocomplexes formed by human antibodies naturally reactive to mouse antibodies, which simultaneously blocked soluble complement inhibitors thus leading to unrestricted complement activation in fluid phase and lowering the amount of complement proteins attainable at the target. To this end, we detected human IgG and IgM reactive to mouse antibodies in our depleted sera (data not shown). In contrast to DGVB buffer, complement activation on mouse/human immunocomplexes is not possible in Mg^++^EGTA buffer and, because of this, the blocking of soluble inhibitors in alternative convertase assays would only increase the pool of complement available to the target. While dissecting the role of particular soluble complement inhibitors in the decay of alternative convertases, C4BP had no significant effect, in contrast to FI and FH (Fig. 4c). Interestingly, blocking of FH alone resulted in much higher readout comparing to the blocking of all soluble inhibitors suggesting an antagonistic effect of blocking antibodies (Fig. 4c).

**Figure 5:**
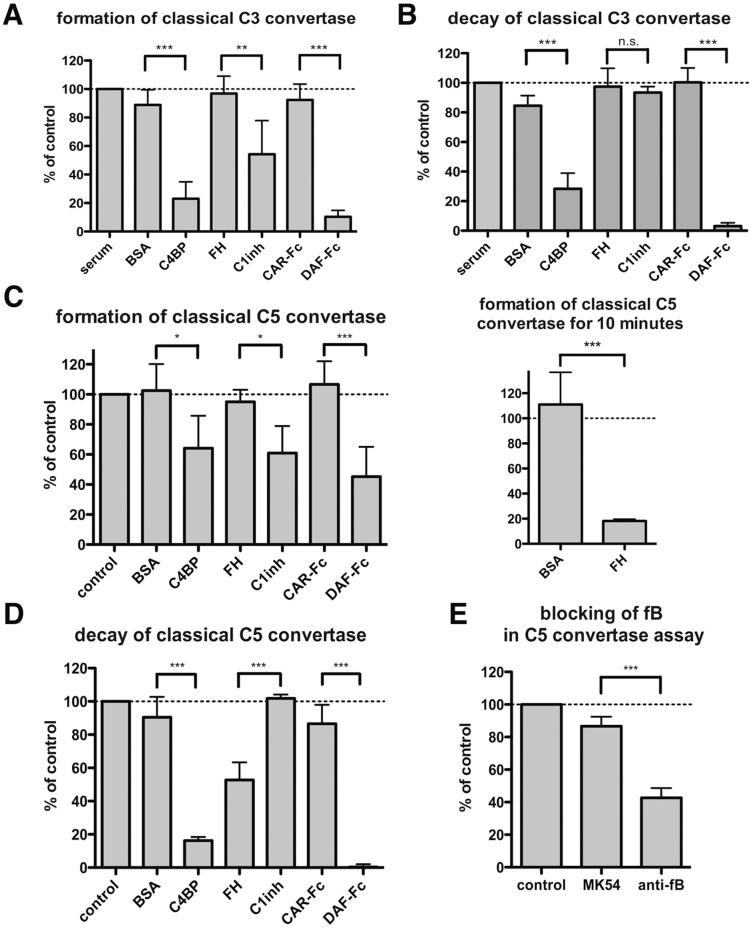
Validation of classical convertase assays with acknowledged complement inhibitors. The classical C3 convertase was formed on the surface of EA with 0.5% of the C3-depleted serum for 5 minutes in the presence (A) or absence (B) of complement inhibitors or control proteins. Then complement mediated lysis was developed with guinea-pig serum in buffer containing EDTA (A) or subjected to another incubation, where dissociation of the convertase was allowed for additional 10 minutes, followed by washing and addition of guinea-pig serum/EDTA (B). Panels C and D present similar assays performed for the classical C5 convertase, which was formed for 2.5 minutes. The insert in panel C shows the effect of FH and BSA on classical C5 convertase formation for 10 minutes. Panel E shows the hemolysis of EA upon blockade of factor B function either at the time of formation or 10 minutes after C5 classical convertase formation. Values were collected from at least three independent experiments and related to control readout (no protein added to C3/C5 -depleted serum). Symbols *, ** and *** stand for p<0.05 or p<0.001, according to one-way ANOVA.

### Functional Assays on Complement Inhibitors

In order to validate our novel convertase assays, we tested several known complement inhibitors with well recognized modes of action. We chose C4BP, FH, FI, C1 inhibitor and the recombinant, extracellular complement control protein (CCP)1-4 domains of human DAF expressed as a Fc-tagged protein together with negative controls: BSA and CAR-Fc. Factors which act via the convertase decay-acceleration mechanism (e.g. C4BP, DAF-Fc and FH in the alternative pathway) should diminish the readout in both convertase formation and decay-acceleration assays, since these take place simultaneously. On the other hand, factors that inhibit complement at early stages via mechanisms different than acceleration of the convertase decay (e.g. C1 inhibitor, FI), should only be effective in convertase formation assays. The mode of action as well as the expected outcome of complement inhibitors used in our assays are summarized in Table 2. Our assumptions were entirely confirmed in assays investigating the classical C3 convertase, C4b2a, formed in C3-depleted serum. C1 inhibitor displayed activity only in the convertase formation assay, whereas C4BP and DAF-Fc were effective in both the formation and decay assays (Fig. 5a,b). BSA and CAR-Fc had no effect and the same was observed for FH, which supports degradation of C3b (not present in this serum) and dissociates the alternative convertases (which cannot be formed in the C3-depleted serum). Similar results were obtained in assays testing the classical C5 convertase with the exception that FH now inhibited hemolysis in decay-acceleration assays whereas it had no effect in the C5 convertase formation assay (Fig. 5c,d). These results were slightly unexpected because FH is usually classified as an exclusive inhibitor of the alternative pathway. However, the classical C3 convertase accelerates conversion of C3 molecules into C3a and C3b. Then, the generated C3b initiates not only assembly of the classical C5 convertase but also C3 (C3bBb) and C5 (C3bBbC3b) alternative convertases as an amplification loop [37]. Therefore, FH acts as an inhibitor of the alternative convertases, which naturally amplify the classical pathway. To determine how much of the C5 convertase activity was generated by the amplification loop, we assembled C5 convertase on EA at 0.5% serum under blockade of FB function and treated such erythrocytes with FB function blocking antibody for an additional 10 minutes. Indeed, half of the total complement-mediated lysis was found to be dependent on FB activity (Fig. 5e) thus explaining the effect of FH in the classical C5 convertase decay-acceleration assay (Fig. 5d). The lack of an effect of FH at the formation stage was probably due to the short time of assembly (2.5 minutes, as Tmax for C5 classical convertase), which might be too brief for efficient FI/FH supported cleavage of C3b and too short for substantial decay of convertases. Indeed, prolongation of convertase assembly to 10 minutes resulted in significant decrease in readout by FH, as compared to control protein (Fig. 5c insert).

**Table 2:** Expected effect on lytic activity of complement inhibitors validated in the assays.

Complementinhibitor	Mechanism of action	Expected outcome in assays					
		classical C3 convertase		classical C5 convertase		alternative convertase	
		formation assay	decay assay	formation assay	decay assay	formation assay	decay assay
C4BP	cofactor for FI –supported cleavage of C3b and C4b,decay-acceleration of classical and alternative convertases	inhibition	inhibition	inhibition	inhibition	inhibition(not tested)	inhibition (not tested)
FH	cofactor for FI –supported cleavage of C3b, decay-acceleration of alternative convertases	no effect	no effect	noticeableinhibitory effect *, **	noticeable inhibitoryeffect *	inhibition	inhibition
C1 inhibitor	prevents assembly of C1 complex and dissociates already formed C1 complex	inhibition	no effect	inhibition	no effect	no effect (not tested)	no effect (not tested)
DAF	decay-acceleration of classical and alternative convertases	inhibition	inhibition	inhibition	inhibition	inhibition	inhibition
FI	protease capable of cleaving activated complement components C3b and C4b in the presence of cofactor	inhibition (not tested)	no effect (not tested)	Inhibition (not tested)	no effect (not tested)	inhibition	no effect

* the noticeable effect is due to inhibition of the amplification loop supported by the alternative complement pathway,

** see insert in Fig. 5c.

**Figure 6:**
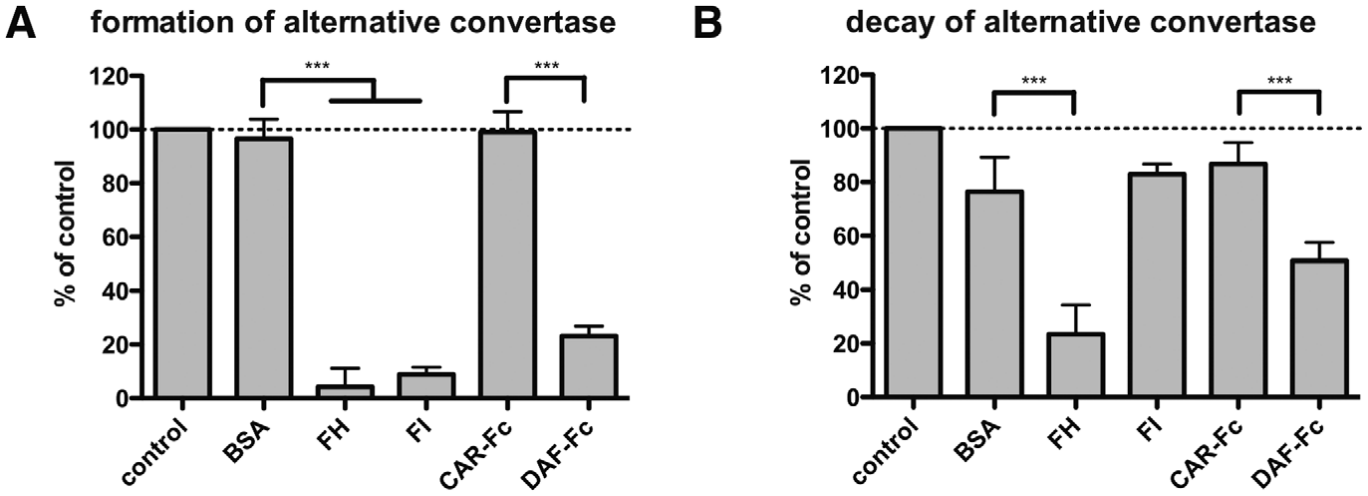
Validation of alternative convertases assays with acknowledged complement inhibitors. Alternative convertases were formed on the surface of rE with 5% of C5-depleted serum for 20 minutes in the presence (A) or absence (B) of complement inhibitors or control proteins. Then complement mediated lysis was developed with guinea-pig serum in buffer containing EDTA (A) or subjected to another incubation, where dissociation of the convertases was allowed for additional 5 minutes, followed by washing and addition of guinea-pig serum/EDTA (B). Values were collected from three independent experiments and related to control readout (no protein added to C3/C5 -depleted serum). Symbols *, ** and *** stand for p<0.05, p<0.01 or p<0.001, respectively according to one-way ANOVA.

For validation of alternative convertase assays, we chose FH (which should be active in both assays), FI (expected to act only at the convertase formation stage) and DAF-Fc (similarly to FH, expected to affect both formation and decay-acceleration of convertases). Unfortunately, we could not try C1 inhibitor as a compound specific only for the classical pathway, since it caused direct lysis of rE, which was visible already after the convertase assembly step (data not shown). We obtained the expected results for all tested compounds (Fig. 6a,b). However, some effect of BSA and FI in decay-acceleration assays could be visible but it did not reach statistical significance when compared to the control value and was clearly distinguishable from the significant effect of active inhibitors.

### The Controversy Regarding Trx-1 Activity on Complement Convertases

**Figure 7:**
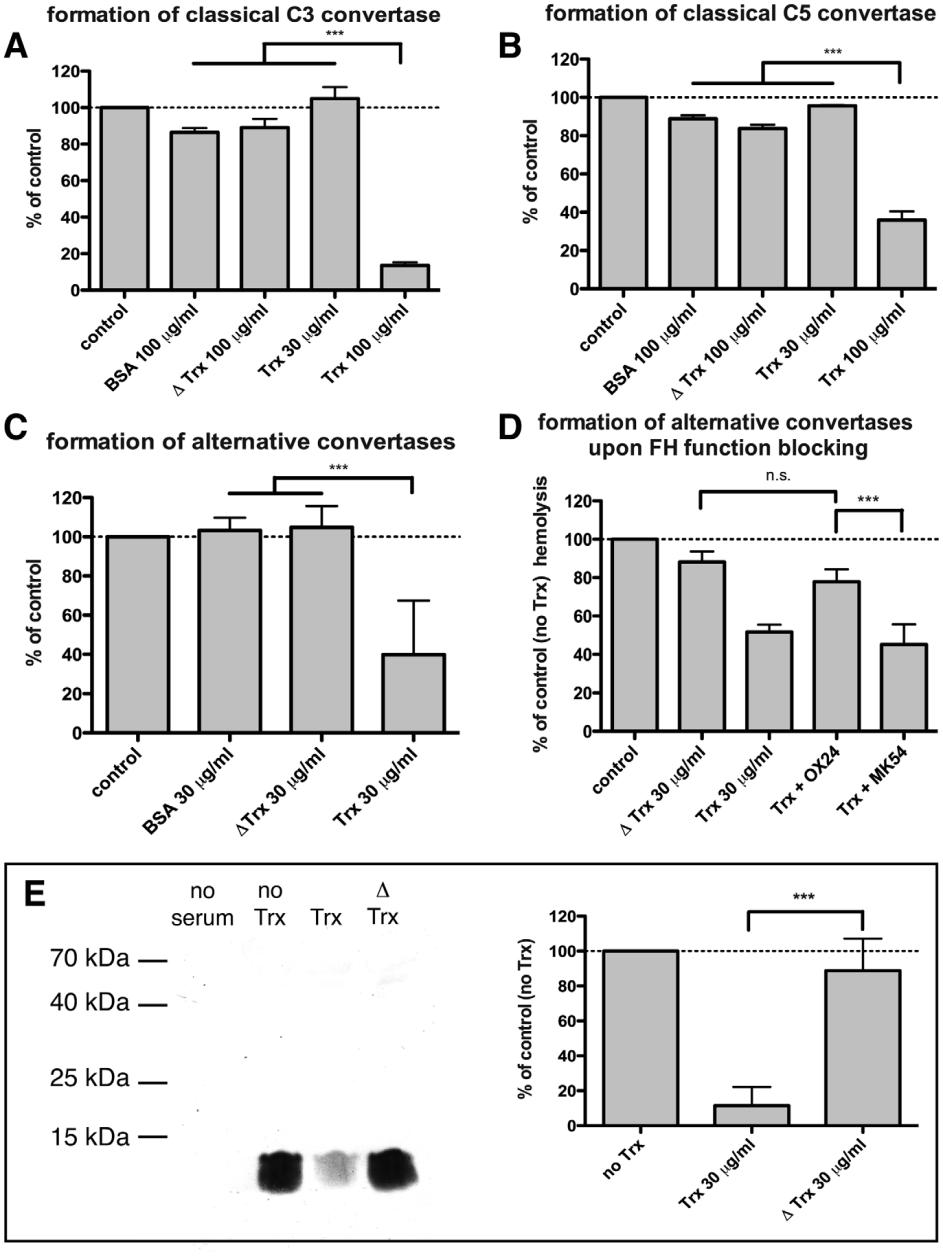
Influence of Trx-1 on convertase formation. Reduced Trx-1, Δ Trx-1 and BSA were tested in classical C3 (A), classical C5 (B) and alternative convertases (C) formation assays. Values were collected from three independent experiments and related to control readout (no protein added to C3/C5 -depleted serum). Panel D shows the effect of Trx-1 on alternative convertases formation upon blockade of FH function by OX24 antibody. Generation of C3a during the treatment of rE with C5-depleted serum with or without Trx-1/ΔTrx-1 is shown in E. The left part shows a representative (out of 4) western blot and the right part shows quantification of the C3a band as percentage of control from all experiments. Symbol *** stands for p<0.001, according to one-way ANOVA.

Trx-1 is a small, redox active protein that possesses a proven complement –inhibitory activity [33], [38], [39]. Complement inhibitory activity is dependent on an enzymatic active site containing reduced cysteine residues, and is proposed to be mediated by interaction with C4BP and FH [33]. However, it is not clear at which stage such inhibition takes place. In order to clarify this issue, we performed convertase formation assays with Trx-1, an active-site mutant Δ Trx-1 and BSA as negative control. Trx-1 but not the mutated form inhibited both C3 and C5 classical convertase formation at 100 µg/ml but was ineffective at 30 µg/ml (Fig. 7 a,b). In contrast, 30 µg/ml of Trx-1 caused significant inhibition of alternative convertases, even though the assay was performed at 10 times higher serum concentration (Fig. 7c). Comparison of the effect of even higher amounts of Trx-1 on the alternative pathway was not possible as the increased amounts of DTT used to reduce the active site of the increased amount of Trx-1, began itself to cause inhibition of complement activation (data not shown). We conclude that Trx-1 is capable of inhibiting all classical and alternative convertases but its effect is more pronounced in inhibition of alternative ones. Next, we wanted to dissect which alternative convertase is primarily affected by Trx-1 and also to examine the reliability of an indirect inhibitory mechanism. Since we found FH as the most relevant soluble inhibitor of alternative convertases (see Fig. 4c), we decided to block its function with OX24 antibody and then test the effect of Trx-1. Indeed, we found that addition of FH -blocking antibody reduced the inhibition of alternative convertases by Trx-1 to a non-significant level (Fig. 7d). Having demonstrated an indirect, FH-dependent mechanism of complement inhibition by Trx-1, a reliable way of determining the alternative convertase primarily affected by Trx-1 was to look for C3a, the activation product of C3, produced by convertases assembled on rabbit erythrocytes. Whereas C3a production was strong in C5 -depleted serum treated with or without 30 µg/ml Δ Trx-1, analogous treatment with Trx-1 resulted in a 90% decrease of C3a (Fig. 7e) indicating that the inhibitory effect of Trx-1 acts already at the level of the C3 alternative convertase.

## Discussion

Since all complement pathways converge at the level of convertases, measuring the convertase activities and how particular components influence them seems to be one of the most important functional assays in the complement field. Nonetheless, there are a limited number of such assays and the existing ones have both advantages and limitations. For example, stepwise assembly of convertases from purified components on the surface of sensitized erythrocytes enables identification of the pure and direct effects of putative inhibitors on convertase functionality, which might be masked and missed in full serum [19], [20]. Also, the old method enables testing of the influence of regulators on a particular step of convertase assembly (e.g. C1 complex activation, processing of C4 or C2), since all stages of convertase formation are separated in time. However, traditionally performed experiments cannot detect the effect mediated by a third protein (for example ones which fix and potentiate another complement inhibitor present in serum). Our serum-based method can detect inhibitors acting both via direct and indirect mechanisms, as we proved for Trx-1, but cannot distinguish between direct and indirect effects on convertases. Nonetheless, such clarification is possible using certain modifications such as neutralisation of the function of the serum protein suspected of being the inhibitory mediator, as we demonstrated for FH in the Trx-1 assay (Fig. 7d). The next problem is how to dissect the activity of a particular convertase in each method. The traditional method employing purified components allows limitation and titration of C3 and thus enables formation of classical C3 (C4b2a) or C5 (C4b2aC3b) convertases on demand. Development of complement-mediated lysis in case of the classical C5 convertase introduces the problem of non-saturated C4b2a, which theoretically may appear in both the traditional and our novel methods in spite of purified C3 addition, or usage of C5-depleted serum sufficient in C3. To evaluate whether such a situation takes place and to assess the activity of only the C5 convertase, the assay should be developed with a mixture of purified C5–C9 components instead of guinea pig serum [29]. However, since we tested MAC development in our assay for the classical C5 convertase with a C5–C9 mix +/− C3 (Fig. 1f) and found only minor activity of the C3 convertase, we concluded that the readouts obtained with guinea pig serum represent mostly C5 convertase activity. Another important issue is the interference of the alternative pathway in classical C5 convertase assays. During the traditional procedure using purified components, formation of alternative convertases (C3bBb or C3bBb3Cb) as an amplification loop is not possible since there is no FB and factor D in the solution. In our experimental setting, formation of alternative convertases, which enhance the effect of the classical C3 convertase, is possible and assessable, as concluded from Fig. 5d, but elimination of this activity is also achievable using FB function blocking antibody (Fig. 5e). More problems occur at the point of distinguishing the activities of alternative C3 and C5 convertases. The traditional method offers titration of C3 which should theoretically saturate all binding sites with a single C3b but still there is no guarantee that the classical convertase employed to deposit C3b on the cell surface does not produce C3b dimers, and thus the alternative C5 convertase is formed prematurely in the next step when FB and factor D are added [19], [29]. One can only evaluate the contribution of alternative C5 convertase activity in the alternative C3 convertase assay by developing it with a C5-C9 mix +/− C3 and comparing the readouts. We have tried such experiments previously and found that lysis developed with C3+C5-C9 was 2.5 times more efficient as compared to only C5-C9 [29], indicating that there is a window to measure the alternative C3 convertase activity in spite of the fact that the final readout would include activities of both convertases (data not shown). Also, during the formation of alternative convertases by the traditional method there is always a C4b molecule (originating from the classical convertase used to deposit C3b) covalently attached to the cell surface in close vicinity to the assembled alternative pathway complex, and thereby acting as a potential steric hindrance for larger ligands. Nonetheless, whereas the traditional method of alternative convertase assembly offers a limited control over the formation of C3bBb or C3bBbC3b, the method using C5-depleted serum gives no opportunity to control such processes. However, conclusions can be made if C3 and C5 cleavage products are measured in the same serum sample following our alternative convertase assay. We demonstrated this technique when C3a was analyzed in the samples collected from rabbit erythrocytes treated with C5–depleted serum with or without Trx-1 (Fig. 7e). Experiments performed on Trx-1 were practical examples of how our new method is useful in solving controversial issues. Recently, we published results showing that Trx-1 causes inhibition of all complement pathways at the level of C5 [33]. We did not observe any effect on C3 deposition under our experimental conditions but a reduced activity of the alternative pathway C3 convertase by Trx-1 was shown by others [38], [39]. Interestingly, Trx-1 was not found to interact directly with complement convertases components (C2, C4, C3, FB) but it does interact with C4BP and FH via its enzymatic active site [33]. Such interaction with complement inhibitors could possibly fix them to, or increase their affinity for, their substrates and explain the complement-inhibitory effect of Trx-1. However, it was not clear why Trx-1 should act specifically on the classical C5 convertase (C4b2aC3b) but not on the classical C3 convertase (C4b2a), since C4BP supports decay-acceleration of both classical convertases as well as cleavage of C4b. The same is true for the alternative pathway, as FH affects both alternative convertases and supports deactivation of C3b. Now we demonstrated a much stronger effect of Trx-1 on alternative than on classical convertases (Fig. 7a,b,c), and since most of the classical C5 convertase activity is supported by the alternative pathway amplification loop (Fig. 3e) some assays might detect additive effects of Trx-1 at the level of C5 but not C3. This could be the reason for the more sensitive detection of classical C5 convertase inhibition found previously, using more traditional whole serum lytic assays [33]. To summarize this, we propose our novel method as an alternative one, which complements the traditional, stepwise formation of convertases from purified components. This procedure is much faster, more affordable and may even detect indirect but physiological inhibitory effects on convertases where the traditional method fails. On the other hand, more detailed insight into direct protein-convertase interaction is offered by the old method and therefore we propose the use of C3 and C5 depleted sera for general screening of possible effects on complement convertases. Also, our method gives a possibility to assess whether complement blockade by a putative inhibitor takes place at early or late stages of the complement cascade before a more detailed investigation, since all effects at the early stages would influence subsequent convertase formation.

Based on our experience with setting up the method, we would like to share some observations applicable for general use. One has to keep in mind, that sequestering C3 or C5 in serum stops the complement cascade at a certain stage but also gives more time for soluble complement inhibitors to act. Therefore it is essential not to extrapolate the *Tmax* from one series of experiments to another but to assess *Tmax* every time that a new batch of depleted serum is used, since there might be substantial differences in complement activators and inhibitors. For example, our Fig. 2 was aimed at providing a general idea about the alternative pathway kinetics and dependencies between time, serum concentration, *Tmax* and activity of complement inhibitors. However, when the series of experiments presented in Fig. 4 were performed with a new bath of C5-depleted serum we found the optimal time of convertase assembly to be 20 minutes instead of 30 minutes, as suggested by the *Tmax* reported in Fig. 2. Moreover, one should provide a validity control for C3 and C5 convertase assays by testing the functionality of C3/C5 depletion from serum (e.g. as shown for C3 and C5-depleted sera in Fig. 2). The natural consequence of C3 or C5 leftovers would be the lysis of erythrocytes visible at the first washing step in functional assays but it might be missed especially when the time of convertase formation is short. Another important validity criteria is assessment of the contribution of the classical C3 convertase activity in the classical C5 convertase assay (as shown in Fig. 1f) and if found to be unsatisfactory at *Tmax*, one can try to optimize the time of convertase formation, serum concentration or development of lysis with a C5–C9 mix. In order to eliminate artifacts in functional assays, a washing step must be performed each time when the solution containing putative complement is changed to the one containing guinea pig serum. Carrying over inhibitors to the lytic developing solution may result in a false positive effect due to the interaction of the added inhibitor with guinea pig serum. We noticed and realized such an effect when studying FH activity in classical C3 convertase formation. Unexpectedly FH caused a decrease of hemolysis and this effect was absent when a washing step was introduced implying that FH did not affect the classical C3 convertase but exerts cofactor activity and decay-acceleration activity in guinea pig serum. Logically, the same precaution must be valid for the traditional method. Since our novel method can not be used to distinguish between the activities of alternative C3 and C5 convertases, it is applicable only for general screening and final conclusions regarding the inhibition of a particular alternative convertase can only be made based on methods allowing C3b titration or by carrying out supporting experiments such as assessment of C3a production.
